# Anesthetic management of patients undergoing mediastinal mass operation

**DOI:** 10.3389/fsurg.2022.1033349

**Published:** 2022-10-28

**Authors:** Jie-chao Tan, Pei-shuang Lin, Li-xian He, Yong Lin, Yun-tai Yao, the Evidence in Cardiovascular Anesthesia(EICA) Group

**Affiliations:** ^1^ Department of Anesthesiology, Fuwai Hospital, National Center for Cardiovascular Diseases, Chinese Academy of Medical Sciences and Peking Union Medical College, Beijing, China; ^2^ Department of Anesthesiology, Shunde Hospital of South Medical University, Foshan, China; ^3^ Department of Anesthesiology, Fujian Medical University Affiliated First Quanzhou Hospital, Quanzhou, China; ^4^ Department of Anesthesiology, Fuwai Yunnan Cardiovascular Hospital, Kunming, China; ^5^ Department of Cardiovascular Surgery, Fujian Medical University Union Hospital, Fuzhou, China

**Keywords:** mediastinal mass, anesthetic management, complications, risk stratificacion, airway management

## Abstract

**Objectives:**

To summarize the anesthetic management of patients undergoing mediastinal mass operation.

**Methods:**

Electronic databases were searched to identify all case reports of patients undergoing mediastinal mass operation. Information such as clinical characteristics, perioperative management and patients’ outcomes were abstracted and analyzed.

**Results:**

Seventy-seven case reports with 85 patients aging from 34 days to 81 years were included. Mediastinal masses were located in anterior (*n* = 48), superior (*n* = 15), middle (*n* = 9) and posterior (*n* = 9) mediastinum, respectively. Clinical manifestations included dyspnea (*n* = 45), cough (*n* = 29), chest or radiating pain (*n* = 12), swelling (*n* = 8), fever (*n* = 7) and chest distress (*n* = 4). Most patients (*n* = 75) had signs of compression or invasion of vital structures. General anesthesia (*n* = 76) was the most commonly used method of anesthesia. Muscle relaxants were administered in 35 patients during anesthesia induction and spontaneous respiration was maintained in 37 patients. Mediastinal mass syndrome (MMS) occurred in 39 cases. Extracorporeal circulation was utilized in 20 patients intraoperatively. Three patients experienced cardiac arrest after ventilation failure and two patients died intraoperatively and one postoperatively.

**Conclusions:**

Peri-operative management of patients undergoing mediastinal mass operation could be challenging. Pre-operative multi-disciplinary discussion, well-planned anesthetic management and pre-determined protocols for emergency situations are all vital to patient safety.

## Introduction

Peri-operative management of patients undergoing mediastinal mass operation could be challenging. Mediastinal mass syndrome (MMS), initially described by Bittar in the 1970 s, is caused by a mediastinal mass, which can quickly deteriorate to acute respiratory and hemodynamic decompensation and is associated with increased morbidity and mortality (1, 2). Therefore, thorough pre-operative assessment, meticulous intra-operative management and multi-disciplinary collaboration are essential when managing patients undergoing mediastinal masses operation (3). Due to absence of guidelines, we performed a literature review of relevant published case reports, to summarize the clinical characteristics, anesthetic management and outcomes of patients undergoing mediastinal mass operation.

## Materials and methods

### Search strategy

Relevant case reports were identified through computerized searches of PubMed, Embase and Ovid databases until May 15th, 2020, using different combinations of search terms “mediastinal mass”, “anesthesia” and “case” (Appendix). Chinese database CNKI was also searched (from the inception to May 15th, 2020). Databases search was updated on August 12th, 2020. Two authors (J.C.T. and P.S.L.) independently reviewed the titles and abstracts of all identified reports for eligibility, with obviously ineligible ones excluded. The eligibility of those remaining reports for final inclusion was determined further by examining the full text. Exclusion criteria included the following: (1) review articles, (2) animal studies, (3) duplicate publications, (4) studies lacking outcomes of interest.

### Data abstraction

The following data from the included case reports were abstracted to a data collection form by two authors (J.C.T. and P.S.L.) independently: (1) literature information (author and year of publication); (2) patients characteristics (age, sex); (3) mediastinal mass features (location, size, pathology) and clinical manifestations (symptoms, signs and examination findings); (4) perioperative management (anesthesia techniques, extracorporeal circulation preparation) and (5) patients' outcomes. Disagreements were resolved by discussion among all authors during the process of data abstraction.

## Results

**Figure 1:**
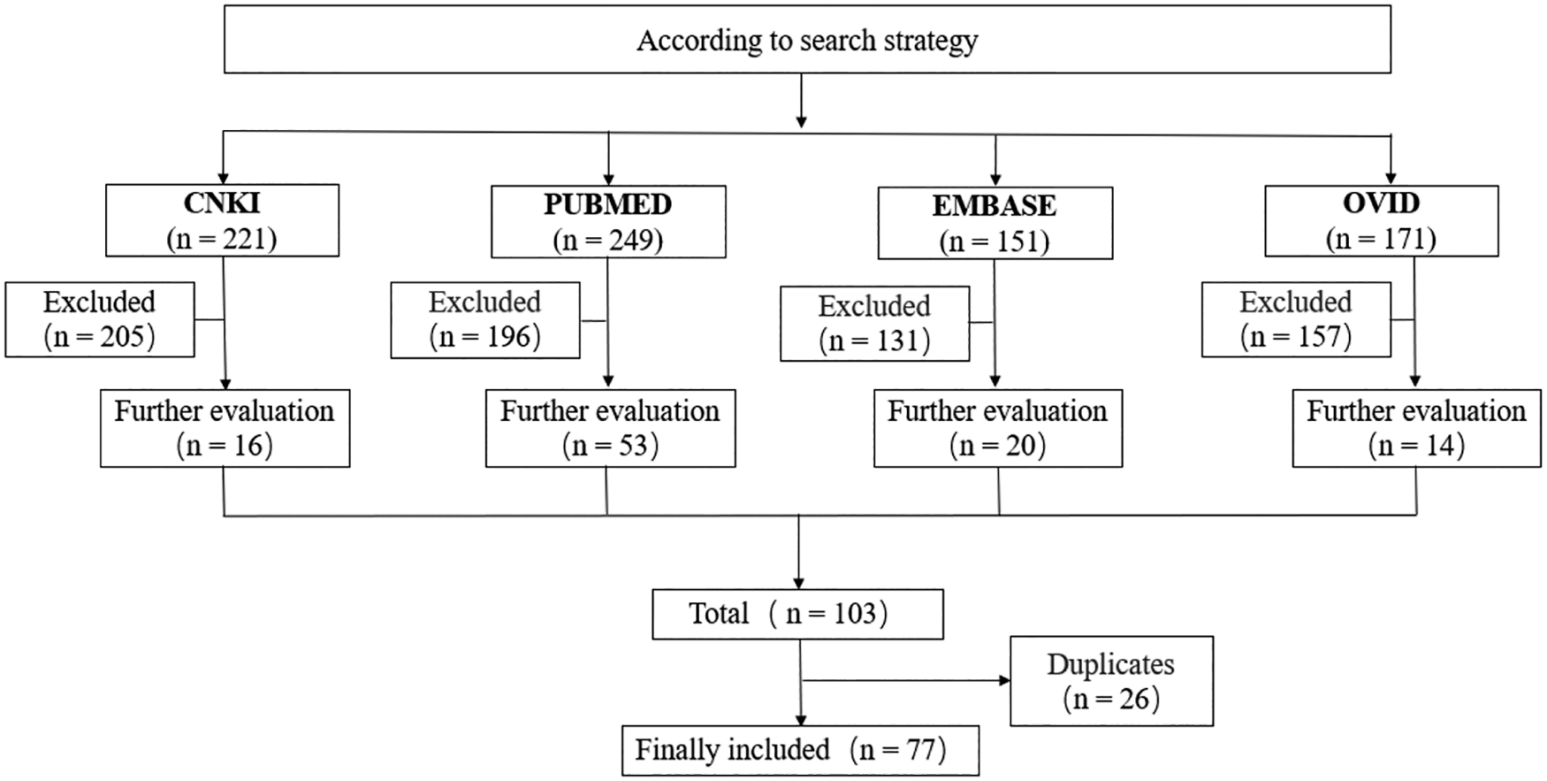
Flow diagram of study selection.

As depicted in the flow chart (Figure 1), the database search identified 103 potentially qualified articles. Seventy-seven case reports (85 patients in total) were determined eligible and included, 66 of which were written in English and the other 11 in Chinese. Descriptive analyses of these cases were presented in Table 1 (4–80).

**Table 1:** Patient characteristics and perioperative data.

Case	Sex	Age	Symptoms and signs	Preoperative examination				MDT	Mediastinal mass			Supine	Compression or invasion				Risk	MMS	Outcomes
				CT	MRI	Echo	PFT		Location	Size (mm)	Pathology		Trachea	Bronchia	SVC	Heart			
Yang 2020	M	65 year	Cough, chest pain	√	*UKN.*	√	√	*UKN.*	PM	*UKN.*	Pheochromocytoma	Yes	/	/	/	/	L	×	Recovery
Armas 2020	M	28 year	Epigastric pain	√	*UKN.*	√	*UKN.*	*UKN.*	AM	141*64*146	Lymphoma	Yes	/	/	√	√	M	×	Recovery
Liu 2020	F	66 year	Dyspnea	√	*UKN.*	*UKN.*	*UKN.*	*UKN.*	PM	101*74*49	*UKN.*	Yes	√	/	/	/	M	×	Recovery
Mohammad 2019	M	57 year	Asymptomatic	*UKN.*	√	*UKN.*	*UKN.*	*UKN.*	SM	*UKN.*	Pericardial cysts	Yes	/	/	/	/	L	×	Recovery
Young-Il 2019	M	67 year	Wheezing, cough, dyspnea	√	*UKN.*	*UKN.*	√	*UKN.*	SM	*UKN.*	Goiter	Yes	√	√	√	/	H	×	Recovery
Sandeep 2019	M	8 month	Crying in supine position, cyanosis	√	*UKN.*	*UKN.*	*UKN.*	*UKN.*	AM	*UKN.*	Lymphoma	Yes^#^	√	√	/	/	H	√	Recovery
Hartigan 2018	M	69 year	*UKN.*	√	*UKN.*	*UKN.*	*UKN.*	*UKN.*	AM	*UKN.*	Carcinoma	Yes	√	√	√	√	M	*UCE.*	*UKN.*
Dubey 2018	M	17 year	Cough, dyspnea, chest distress, facial swelling	√	*UKN.*	√	√	*UKN.*	AM	*UKN.*	*UKN.*	No	√	√	√	√	H	√	Recovery
Kafrouni 2018	F	30 year	Cough, dyspnea, weight loss	√	*UKN.*	*UKN.*	*UKN.*	*UKN.*	AM	*UKN.*	Lymphoma	Yes	√	/	/	/	M	√	OP cancel
Bruce 2018	M	5 month	Wheezing	√	*UKN.*	√	*UKN.*	√	AM	82*62*73	Teratoma	No	√	√	√	√	H	√	Recovery
Liu 2018	F	5 year	Cough	√	*UKN.*	UKN.	*UKN.*	*UKN.*	PM	111*91*?	Neurogenic tumor	UKN	√	√	√	√	H	√	Recovery
Mitra 2018	F	34 day	Dyspnea	*UKN.*	*UKN.*	√	*UKN.*	*UKN.*	AM	50*45*30	Teratoma	Yes^#^	√	√	√	√	H	*UCE.*	Recovery
Liu 2017	F	54 year	Dyspnea	√	*UKN.*	*UKN.*	√	*UKN.*	SM	61*86*91	Bronchogenic cyst	Yes^#^	√	/	√	√	H	*UCE.*	Recovery
Freed 2017	F	81 year	Dyspnea	√	*UKN.*	√	*UKN.*	*UKN.*	AM	75*47*45	Thymoma	Yes	/	/	√	√	L	√	Recovery
Saipriya 2017	F	14 year	Dyspnea	√	*UKN.*	*UKN.*	*UKN.*	*UKN.*	PM	140*35*40	Enteric cyst	Yes^#^	√	√	/	/	H	*UCE.*	Recovery
Kusajima 2017	F	30 year	Cough, dyspnea	√	*UKN.*	*UKN.*	*UKN.*	√	AM	113*87*68	Lymphoma	No	√	√	√	/	H	×	Recovery
Juan 2017	F	44 year	Asymptomatic	√	*UKN.*	*UKN.*	*UKN.*	*UKN.*	PM	50*64*?	Pheochromocytoma	Yes	*UKN.*	*UKN.*	*UKN.*	*UKN.*	L	×	Recovery
Nokes 2017	F	49 year	Dyspnea	√	*UKN.*	*UKN.*	*UKN.*	*UKN.*	*UKN.*	*UKN.*	*UKN.*	Yes^#^	√	/	√	/	H	×	Recovery
Wang 2017	F	56 year	Dysphagia	√	*UKN.*	*UKN.*	*UKN.*	*UKN.*	SM	200*150*60	Goiter	Y	√	/	/	/	M	×	Recovery
Ayşe 2017	F	6 month	Dyspnea	√	*UKN.*	*UKN.*	*UKN.*	*UKN.*	AM	93*78*?	Teratoma	Yes^#^	√	√	/	/	H	√	Recovery
Sulen 2016	F	36 year	Cough, chest pain, dyspnea	√	*UKN.*	*UKN.*	*UKN.*	*UKN.*	PM	80*75*53	Bronchogenic cyst	Yes^#^	√	/	/	/	H	×	Recovery
Dudley 2016	M	28 year	Cough, dyspnea, sore throat, neck swelling	√	*UKN.*	*UKN.*	*UKN.*	*UKN.*	AM	*UKN.*	Seminoma	No	√	√	√	/	H	*UCE.*	Recovery
Maria 2016	F	11 year	Facial and arm swelling	√	*UKN.*	√	*UKN.*	√	AM	100*80*?	Lymphoma	*UKN.*	√	√	√	/	M	√	Recovery
Wooles 2015	M	59 year	Chest pain, hoarseness, dysphagia	√	*UKN.*	*UKN.*	*UKN.*	√	SM	*UKN.*	Bronchogenic cyst	No	√	/	√	/	H	√	Recovery
Scheele 2015	M	23 year	Dyspnea	√	*UKN.*	*UKN.*	*UKN.*	√	AM	150*100*160	Seminoma	No	√	√	√	/	H	√	Recovery
Ghada 2015	M	14 year	Dyspnea	√	*UKN.*	*UKN.*	*UKN.*	*UKN.*	MM	35*44*42	Lipoma	Yes^#^	√	√	√	/	H	√	Recovery
Lee 2014	F	35 year	Asymptomatic	√	*UKN.*	*UKN.*	*UKN.*	*UKN.*	AM	75*92*14	Teratoma	Yes	/	/	/	/	L	√	Recovery
Thakur 2014	F	4 year	Cough	√	*UKN.*	*UKN.*	*UKN.*	*UKN.*	SM	57*46*28	Bronchogenic cyst	Yes^#^	√	/	/	/	M	×	Recovery
Rajagopalan 2014	F	64 year	Dyspnea, cough, hoarseness of voice	√	*UKN.*	*UKN.*	*UKN.*	*UKN.*	AM	103*104*127	Carcinoma	Yes^#^	√	√	√	√	H	×	Recovery
Said 2014	F	37 year	Dyspnea	√	*UKN.*	√	*UKN.*	*UKN.*	AM	170*90*120	*UKN.*	No	√	√	√	√	H	√	Recovery
Brain 2014	M	65 year	Fever, hypoxemia	√	*UKN.*	√	*UKN.*	√	MM	50*40*40	Lymphoma	Yes	/	/	√	/	M	×	Recovery
Michael 2014	M	24 year	Fatigue, fever, hoarseness, cough, dyspnea	√	*UKN.*	*UKN.*	*UKN.*	√	AM	*UKN.*	Lymphoma	Yes^#^	√	√	√	√	H	*UCE.*	Recovery
Ward 2014	F	39 year	Cough, shoulder pain, dyspnea	√	*UKN.*	*UKN.*	*UKN.*	*UKN.*	AM	*UKN.*	Thymoma	No	√	√	√	√	H	*UCE.*	Recovery
Ibrahim 2013	F	21 year	Cough, dyspnea	√	*UKN.*	*UKN.*	*UKN.*	*UKN.*	AM	*UKN.*	*UKN.*	Yes^#^	√	√	√	√	H	√	Death
Chrystelle 2013	M	11 year	Dyspnea	√	*UKN.*	*UKN.*	*UKN.*	*UKN.*	AM	98*145*156	Lymphoma	Yes^#^	√	√	√	√	H	×	Recovery
Chrystelle 2013	M	15 year	Weight loss	√	*UKN.*	√	*UKN.*	*UKN.*	SM	*UKN.*	Lymphoma	Yes^#^	√	√	√	/	H	×	Recovery
Lalwani 2013	M	23 year	Dyspnea, hoarseness	√	√	√	*UKN.*	*UKN.*	PM	130*150*130	Carcinoma	Yes	√	√	√	/	H	*UCE.*	Recovery
Rim 2013	F	60 year	Dyspnea	√	*UKN.*	*UKN.*	*UKN.*	*UKN.*	MM	73*59*61	Schwannoma	Yes	√	√	√	√	M	×	Recovery
Miyauchi 2013	M	15 year	Asymptomatic	√	*UKN.*	*UKN.*	*UKN.*	*UKN.*	AM	160*140*130	Teratoma	Yes	/	√	√	√	M	×	Recovery
Han 2013	F	15 year	Ulceration	√	*UKN.*	√	*UKN.*	*UKN.*	PM	60*60*50	Paraneoplastic pemphigus	Yes	*UKN.*	*UKN.*	*UKN.*	*UKN.*	M	×	Recovery
Peter 2012	F	57 year	Neck swelling	√	*UKN.*	*UKN.*	*UKN.*	√	SM	*UKN.*	*UKN.*	Yes	√	/	/	/	H	×	Recovery
Chaudhary 2012	M	42 year	Face, neck, chest and upper arms swelling	√	*UKN.*	*UKN.*	*UKN.*	*UKN.*	AM	*UKN.*	Lymphoma	Yes	√	/	√	/	M	√	Recovery
John 2012	F	9 year	Cough, dyspnea, facial swelling	√	*UKN.*	√	*UKN.*	√	AM	*UKN.*	Carcinoma	No	√	√	√	√	H	×	Recovery
Gautam 2012	M	1 month	Stridor, dyspnea, cyanosis, periorbital edema	√	*UKN.*	√	*UKN.*	*UKN.*	AM	50*50*?	Teratoma	Yes^#^	√	√	√	/	H	×	Recovery
Yao 2012	F	59 year	Chest distress	√	*UKN.*	*UKN.*	√	*UKN.*	AM	200*200*15	*UKN.*	Yes^#^	√	√	√	√	H	√	Recovery
Gardner 2011	M	19 year	Cough, fever, weight loss	√	*UKN.*	*UKN.*	*UKN.*	*UKN.*	AM	*UKN.*	Lymphoma	Yes	√	√	/	/	H	√	Recovery
David 2011	F	23 year	Chest pain, back pain	√	√	*UKN.*	*UKN.*	*UKN.*	AM	49*76*58	Lymphoma	Yes	/	/	/	/	M	√	Recovery
Benedicte 2011	F	4 year	Dyspnea	√	*UKN.*	*UKN.*	√	*UKN.*	AM	180*11*80	Fibromatosis	No	√	/	√	/	H	×	Recovery
Betina 2011	F	14 year	Cough, dyspnea	√	*UKN.*	*UKN.*	*UKN.*	*UKN.*	AM	*UKN.*	Lymphoma	Yes^#^	√	√	√	√	H	×	Recovery
Woo 2010	M	18 year	Cough, dyspnea, chest distress	√	*UKN.*	√	√	*UKN.*	AM	120*90*150	Lymphoma	Yes^#^	√	√	√	√	M	√	Recovery
Chen 2010	F	53 year	Cough, dyspnea	√	*UKN.*	*UKN.*	*UKN.*	*UKN.*	AM	200*100*70	Goiter	Yes	√	/	√	/	M	√	Recovery
Yang 2009	*UKN.*	3 year	*UKN.*	√	*UKN.*	*UKN.*	*UKN.*	*UKN.*	MM	*UKN.*	*UKN.*	*UKN.*	√	√	/	/	M	√	Recovery
Yang 2009	*UKN.*	6 year	*UKN.*	√	*UKN.*	*UKN.*	*UKN.*	*UKN.*	MM	*UKN.*	*UKN.*	*UKN.*	√	√	/	/	M	√	Recovery
Yang 2009	*UKN.*	16 year	*UKN.*	√	*UKN.*	*UKN.*	*UKN.*	*UKN.*	MM	160*160*135	*UKN.*	*UKN.*	√	√	/	/	M	√	Recovery
Yang 2009	*UKN.*	10 year	*UKN.*	√	*UKN.*	*UKN.*	*UKN.*	*UKN.*	MM	*UKN.*	*UKN.*	*UKN.*	√	√	/	/	M	√	Recovery
Mourad 2009	M	42 year	Asymptomatic	√	√	*UKN.*	*UKN.*	√	AM	220*190*170	Thymolipoma	Yes	√	√	/	/	M	×	Recovery
Basem 2009	M	41 year	Dyspnea	√	*UKN.*	*UKN.*	*UKN.*	*UKN.*	AM	100*90*110	Carcinoma	Yes^#^	√	√	√	/	H	×	Recovery
Basem 2009	M	62 year	Cough, dyspnea	√	*UKN.*	*UKN.*	*UKN.*	*UKN.*	AM	*UKN.*	*UKN.*	Yes^#^	√	√	√		H	√	Recovery
Wang 2009	M	72 year	Dyspnea	√	*UKN.*	*UKN.*	*UKN.*	*UKN.*	SM	40*50*70	Goiter	*UKN.*	√	/	/	/	M	×	Recovery
Zhang 2007	F	72 year	*UKN.*	√	*UKN.*	*UKN.*	*UKN.*	*UKN.*	PM	34*40*?	*UKN.*	*UKN.*	√	/	/	/	H	×	Recovery
Zhang 2007	F	56 year	*UKN.*	√	*UKN.*	√	*UKN.*	*UKN.*	AM	35*30*?	*UKN.*	No	√	√	/	/	H	√	Recovery
Frey 2006	M	10 year	Dyspnea, orthopnea, fever	√	*UKN.*	√	*UKN.*	*UKN.*	AM	*UKN.*	Lymphoma	Yes^#^	√	√	√	√	M	×	Recovery
Goppm 2005	F	42 year	Dyspnea, arrhythmia, chest pain, fever	√	*UKN.*	*UKN.*	*UKN.*	*UKN.*	AM	*UKN.*	Enteric cyst	Yes^#^	√	√	√	/	M	×	Recovery
Qu 2005	M	3 year	Dysphagia	√	*UKN.*	√	*UKN.*	*UKN.*	AM	*UKN.*	Enteric cyst	No	√	√	/	/	H	√	Recovery
Yasunori 2004	M	17 year	Dyspnea, tachycardia, hypotension	√	*UKN.*	√	*UKN.*	*UKN.*	AM	*UKN.*	Lymphoma	Yes^#^	√	√	√	√	H	√	Recovery
Dilworth 2003	M	9 year	Jugular vein distention, cough, dyspnea,	√	*UKN.*	√	*UKN.*	*UKN.*	MM	*UKN.*	Lymphoma	No	√	/	√	/	H	×	Recovery
Li 2003	M	3 month	Cyanosis	√	*UKN.*	*UKN.*	*UKN.*	*UKN.*	AM	*UKN.*	Enteric cyst	Yes^#^	/	√	/	/	M	×	Recovery
Dilworth 2001	M	15 year	Cough, dyspnea	√	*UKN.*	√	*UKN.*	*UKN.*	SM	*UKN.*	Lymphoma	No	√	√	√	√	H	√	Recovery
Tempe 2001	M	22 year	Chest and neck pain, dyspnea, neck swelling	√	*UKN.*	*UKN.*	√	*UKN.*	AM	120*100*?	Lipoma	Yes^#^	√	√	√		H	√	Recovery
Shi 2000	F	16 year	Chest distress	√	*UKN.*	*UKN.*	*UKN.*	*UKN.*	*UKN.*	200*200*180	*UKN.*	*UKN.*	√	√	√	√	M	√	Recovery
Vas 1999	M	2 month	Crying, cough, dyspnea	√	*UKN.*	*UKN.*	*UKN.*	*UKN.*	SM	*UKN.*	*UKN.*	No	√	√	√	/	H	√	Recovery
Licker 1997	F	47 year	Dyspnea, hemoptysia, fever	√	*UKN.*	*UKN.*	√	*UKN.*	SM	*UKN.*	Carcinoma	Yes^#^	√	/	/	/	M	×	Recovery
Goh 1999	F	20 year	Cough	√	*UKN.*	*UKN.*	*UKN.*	*UKN.*	AM	*UKN.*	Lymphoma	Yes	√	√	√	/	M	√	Recovery
Furst 1996	F	9 year	*UKN.*	√	*UKN.*	*UKN.*	*UKN.*	*UKN.*	SM	*UKN.*	Vascular malformation	Yes^#^	/	/	√	/	M	×	Recovery
Polaner 1996	M	3 year	Dyspnea	√	*UKN.*	*UKN.*	*UKN.*	*UKN.*	AM	*UKN.*	Lymphoma	No	√	√	/	/	H	×	Recovery
Hattamer 1996	F	29 year	Cough, dyspnea	√	*UKN.*	*UKN.*	√	*UKN.*	PM	150*80	Lymphoma	Yes^#^	/	/	/	/	M	×	Recovery
Frawley 1995	M	13 year	Cough	√	*UKN.*	*UKN.*	*UKN.*	*UKN.*	AM	*UKN.*	*UKN.*	Yes^#^	√		√	/	M	×	Recovery
Susheela 1995	M	8 year	*UKN.*	√	√	√	*UKN.*	*UKN.*	SM	31*50*28	Lymphoma	Yes	*UKN.*	*UKN.*	*UKN.*	/	M	√	Death
Wang 1995	F	28 year	Cough, chest pain	√	*UKN.*	*UKN.*	*UKN.*	*UKN.*	*UKN.*	340*180*80	Teratoma	Yes	√	√	√	√	M	√	Recovery
Montange 1994	F	12 year	Asymptomatic	√	*UKN.*	*UKN.*	*UKN.*	*UKN.*	SM	*UKN.*	Lymphoma	Yes	√	/	/	/	L	√	Recovery
John 1988	M	12 year	Jugular vein distention, dyspnea	√	*UKN.*	*UKN.*	*UKN.*	*UKN.*	*UKN.*	*UKN.*	Lymphoma	Yes	√	/	/	/	M	√	Recovery
Prakash 1988	M	24 year	Cough, neck and shoulder pain	√	*UKN.*	√	*UKN.*	*UKN.*	AM	*UKN.*	Lymphoma	Yes^#^	√	√	√	√	M	√	Recovery
Neuman 1984	M	13 year	Cough, dyspnea, facial swelling	√	*UKN.*	*UKN.*	*UKN.*	*UKN.*	AM	*UKN.*	Lymphoma	No	/	/	√	/	H	√	Death
Neuman 1984	M	16 year	Cough, dyspnea, chest pain, night sweats	√	*UKN.*	*UKN.*	*UKN.*	*UKN.*	AM	*UKN.*	Lymphoma	Yes^#^	/	/	/	/	M	×	Recovery
Neuman 1984	M	13 year	Chest pain, fever	√	*UKN.*	*UKN.*	*UKN.*	√	AM	*UKN.*	Lymphoma	Yes	/	/	/	/	M	×	Recovery

AM, anterior mediastinum; d, day; F, female; LV, left ventricle; M, male; m, month; MM, middle mediastinum; MMS, middle mediastinum syndrome; OP, operation; PM, posterior mediastinum; SM, superior mediastinum; SP, supine position; SVC, superior vena cava; UCE, uncertain; UKN, unknown; Yes^#^, Yes but with symptoms and signs; y, years.

The 85 patients aged between 34 days and 81 years, of whom 42 were males and 39 females (4 cases did not describe sex). Forty-eight (59.3%) cases of masses were located in anterior mediastinum, 15 (28.5%) in superior mediastinum, 9 (11.1%) in middle mediastinum and 9 (11.1%) in posterior mediastinum. The mass sizes ranged from 35mm × 44mm × 42 mm to 200mm × 200mm × 180 mm. Anterior mediastinum masses were usually bigger than those of other origins. Lymphoma (28/85, 32.9%) was the most common pathological type, followed by teratoma or seminoma (9/85, 10.5%). Of the 85 patients, 45 (59.2%) presented with dyspnea, 29 (38.1%) with cough, 12 (15.8%) with chest or radiating pain, 8 (10.5%) with swelling, 7 (9.2%) with fever, 4 (5.2%) with chest distress and 6 (7.8%) patients were asymptomatic. Seventy-five cases had signs of compression or invasion of trachea (60/85, 70.6%), bronchia (55/85, 64.7%), superior vena cava (43/85, 50.6%) and heart (26/85, 30.6%). Examination such as Computed tomography (CT), transthoracic echocardiogram (TTE), pulmonary function testing (PFT) and magnetic resonance imaging (MRI) were performed in 84 (97.4%), 24 (28.2%), 10 (11.8%), and 5 (5.9%) patients, respectively. Fifty-one (60.0%) patients underwent open thoracotomy, 28 (32.9%) patients underwent video-assisted thoracoscopic surgery (VATS) and 6 (7.1%) patients underwent other surgery. Fifty-seven (67.1%) patients had mass resection, 27(31.8%) patients received mass biopsy and 1(1.1%) case did not report surgical procedure.

Seventy-six (89.4%) patients were operated under general anesthesia (GA), 8 (9.4%) patients under sedation and 1 (1.2%) patient under local anesthesia. Fentanyl (*n* = 23), midazolam (*n* = 21), propofol (*n* = 19), ketamine (*n* = 15) and sevoflurane (*n* = 12) were most frequently used agents, followed by dexmedetomidine (*n* = 6), halothane (*n* = 5), remifentanil (*n* = 4), etomidate (*n* = 3), nitrous oxide (*n* = 3), diazepam (*n* = 2) and isoflurane (*n* = 2). Muscle relaxants were reported to be administered in 35 of the 85 included patients during anesthesia induction and in 5 patients after sternotomy, respectively. Succinylcholine (9/20, 45%) was the most commonly used muscle relaxant before endotracheal intubation. As for airway management, 66 (77.6%) patients were intubated with single lumen tube (SLT) including 1 with bronchial blocker (BB), 5 (5.9%) with double lumen tube (DLT), 4 (4.7%) with laryngeal mask airway (LMA) and 9 (10.6%) patients were tubeless. Spontaneous respiration was maintained in 32 (37.6%) patients, including 23 with spontaneous ventilation (SV) and 9 with assisted ventilation (AV).

Thirty-nine (45.9%) included patients developed MMS, 2 (2.4%) cases occurred before anesthetic induction, 13 (15.3%) cases after non-paralytic (without muscle relaxant) endotracheal intubation, 3 (3.5%) cases after muscle relaxant administration, 10 (11.8%) cases during position change, 10 (11.8%) cases during mass dissection, 3 (3.5%) cases during post-anesthesia recovery, respectively. Extracorporeal circulation (ECC) technique was applied in 20 (23.5%) patients: 2 initiated before anesthesia induction and 18 just with ECC standby. Three patients underwent ECC support due to severe intraoperative cardiopulmonary collapse. One patient experienced severe oxygen desaturation as the airway collapsed after endotracheal intubation without muscle relaxant and the operation was finally cancelled (12). Three patients died: 2 patients died from cardiopulmonary arrest as a result of ventilation failure during the anesthesia induction (37, 80); 1 patient had cardiac arrest intraoperatively due to suddenly increased airway resistance which deteriorated to sustained ventilation failure. The patient died on postoperative day 10 (75).

## Discussion

Anesthetic management of mediastinal mass operation could be complicated by MMS characterized by acute respiratory and hemodynamic decompensation, which is caused by mechanical compression of mediastinal structures (81). However, no relevant guidelines for management of patients undergoing mediastinal mass operation is currently available. In the present study, we summarized the clinical characteristics, anesthetic management and outcomes of 85 patients undergoing mediastinal mass operation.

Comprehensive preoperative assessment is crucial in the management of patients with mediastinal mass (14, 39). Some risk stratifications have been proposed based on patients' preoperative signs and symptoms and the degree of major vessel or airway compression (2). CT scan, as an initial choice, provides anatomical details of masses and their relations with surrounding structures and helps in the creation of a deliberate plan for anesthetic and surgical management (28, 34). Compared with CT scan, MRI is more sensitive in soft tissue differentiation and delineating tissue boundaries. Echocardiography evaluates cardiac structural and functional alterations, which might influence anesthetic and surgical decision making (36). PFT, a tool to assess respiratory dysfunction and airway obstruction, may be of less value since there is conflicting evidence regarding the utility of PFT in risk stratification of mediastinal mass patients (7). By integrating the existing data of patients with mediastinal mass (2, 82, 83), we propose a classification to help categorize patients into three categories s of risk (Table 2). A detailed preoperative multidisciplinary team (MDT) discussion is of vital importance (14, 37).

**Table 2:** Risk classification & categories of mediastinal mass patients.

Risk classification	Signs and symptoms	Imaging examination findings		
Safe	−	−		
Unsafe	+	+		
Uncertain	+	−/NA		
	−	+: Tracheobronchial CSA < 50% or compressed heart/great vessel		
Risk categories	Signs and symptoms	Supine tolerability	Tracheobronch	Heart or great vessel
Low	Negative	Yes	Negative	Negative
Medium	Chest distress Dyspnea Swelling Tachycardia	Yes (discomfortable)	Displaced or CSA < 50%	Displaced
High	Cyanosis Orthopnea Stridor SVC syndrome	No	CSA > 50%	Tamponade

Abbreviation: CSA, cross-sectional area; CT, computed tomography; ECHO, echocardiography; MRI, magnetic resonance imaging, NA, not available, SVC, superior vena cava.

Some authors (5, 7, 8, 12, 24, 35, 44, 49, 57) have outlined some suggestions for the anesthetic management of mediastinal mass patients undergoing surgery. For example, avoidance of general anesthesia (especially paralytic agents) or maintenance of SV has been recommended (35, 69). It is a consensus to proceed with stepwise induction and avoid deep sedation (37). It has been agreed that, no single agent is superior to another one, and that any agent should be used judiciously in consideration of retaining SV. Frawley et al. (74) reported a lower incidence of respiratory depression when ketamine was used alone or when combined with midazolam, provided the dose of the latter was kept low (0.1 mg/kg). Propofol can maintain spontaneous respiration when given slowly (40), even though when combined with remifentanil which may result in increased PaCO_2_ (83). Basem et al*.* (56) concluded that maintaining with dexmedetomidine (dose range of 0.2–0.7 µg/kg/h) could be very helpful and may reduce the risk of complete airway obstruction in the anesthetic management of mediastinal mass. Of the 85 patients, although 54(63.5%) did not receive muscle relaxant in induction, the rate of MMS was not lower than that of patients who received muscle relaxant. A retrospective study by Ng et al. (84) found that positive-pressure ventilation and intubation (though no muscle relaxation) was used in all cases that reported complications.

Virtually all reported cases of severe MMS occurred after abolition of SV (1). Dubey (11) suggested that maintaining SV until sternotomy is a safer approach. If a muscle relaxant is required, manually assisted ventilation should be done firstly, to assure that positive-pressure ventilation is possible and then a short-acting muscle relaxant can be administered (12, 24). However, the return of spontaneous breathing is not quick enough in critical situations (2). As a result, we advocate no muscle relaxant and maintenance of SV in anesthetic induction. Of note, maintenance of SV cannot assure airway patency during anesthesia. Gardner (48) considered that partial upper airway obstruction may generate sufficiently negative intraluminal pressure to collapse the compromised segment in the rapidly spontaneously breathing patient, which explains why the dynamic airway collapse and the inability to ventilate despite maintenance of SV.

Opinions about airway management in patients with MMS differed among authors. Kafrouni et al. (12) suggested that both lungs ventilation *via* a reinforced SLT was preferred. In the current study, more than 60% cases were intubated with SLT. Sulen (24) advised that for patients with airway obstruction, the safest option was to place a bronchial blocker (BB) or double lumen tracheal (DLT) tube when patients remained awake. Compared with conventional intubation method, extraluminal use of BB has more advantages (6). DLT intubation was suitable for low risk of airway compression (30). Since the increased availability of fiberoptic bronchoscopy (FOB) in many institutions, awake intubation guided by FOB has become another useful option for airway management in mediastinal masses patients (especially those with airway compression). In Rajagopalan (32) and Miyauchi's view (51), LMA or bi-level positive airway pressure (BiPAP) in sedation anesthesia can be used in patients with mediastinal mass that needs an incisional biopsy while maintaining SV. It is important to adhere to the general principles of maintaining effective ventilation and hemodynamic stability during induction and maintenance of anesthesia regardless of the technique used.

MMS can occur in every stage of perioperative period (2). Airway compression can occur in preoperatively asymptomatic adults with mediastinal mass (48). Acute respiratory decompensation may be precipitated by positional changes (8). Positioning change may help to relieve the mass effect of tumors (31, 37, 43, 46). It is vital to identify comfortable position in terms of respiration and hemodynamics in those symptomatic patients prior to surgery. The right lateral decubitus position can prevent MMS when the sitting position is not effective during general anesthesia (52). When intraoperative MMS does occur due to mass dissection, operation should be paused and compression be relieved immediately (81).

In high-risk patients classified as unsafe, decompensation after anesthesia induction should be expected and the option of connection to an extracorporeal circulation must always be provided (8, 52, 66). In the current study, ECC was prepared in 15 medium to high risk patients and 3 of them completed the operation with ECC when there was a severe cardiopulmonary failure intraoperatively. ECC were established before anesthesia induction and completed the operation successfully in 2 high risk patients. Three patients died of acute respiratory failure without ECC support and 1 patient's operation was canceled because of the cardiopulmonary system unsteadiness, also with an absence of ECC preparation. Tempe et al. (66) cannulated the femoral vessels and kept ECC ready because it was thought that there was a definite danger of the patient developing airway obstruction. Maria et al. (26) and Brandon (21) provided a successful example of ECC used in this context to assist with high-risk MMS patients with impending respiratory collapse. Recently, extracorporeal membrane oxygenation (ECMO) has become popular, which could be utilized as an easy form of ECC supporting circulatory and/or pulmonary functions in high-risk MMS patients.

**Figure 2:**
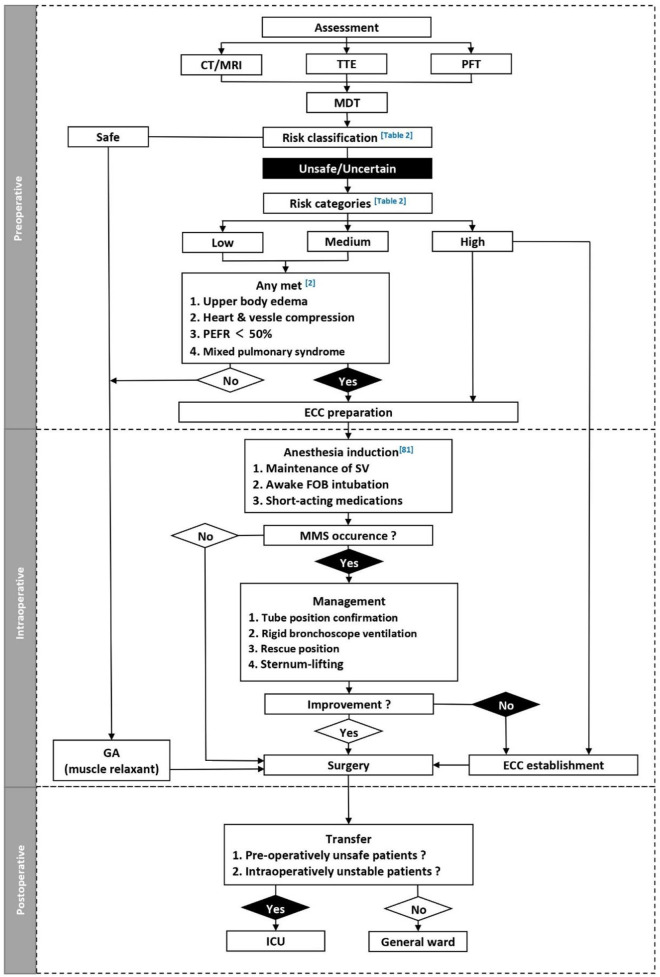
Perioperative management of patients with mediastinal mass. CT, computed tomography; ECC, extracorporeal circulation; FOB, fiber optic bronchoscope; GA, general anesthesia; ICU, Intensive care unit; MDT, multidisciplinary team; MMS, mediastinal mass syndrome; PEFR, peak expiratory flow rate; PFT, pulmonary function testing; SV, spontaneous ventilation; TTE, transthoracic echocardiogram.

Following surgery, patients in the unsafe risk category should be transferred to an intensive care unit (ICU). The extent of postoperative monitoring for patients in the uncertain risk category should be decided depending on preoperative findings and intraoperative course (2). It is worth noting that completion of the operation does not mean that the alert could have been lifted. Unexpectedly, 2 (28, 79) patients experienced MMS in recovery period. One had failed extubation and was transferred to ICU and the other one had tracheal compression with ventilation obstruction. The sitting position is preferable for resuscitation in the presence of an airway obstruction (52). By combining the above-mentioned practice and suggestions, a recommended management protocol (Table 3) and flowcharted (Figure 2) was formed for patients undergoing mediastinal mass operation.

**Table 3:** Recommended management of patients undergoing mediastinal mass operation.

**Preopreative examination and treatment**
CT	High-resolution CT-angiography and multiplanar reconstruction
MRI	Assessment of mediastinum and chest wall invasion
Echocardiography	Evaluation of compression, cardiovascular structure/function and volume status
PFT	Prediction of peri-operative respiratory complications
FDG-PET	Diagnosis, tumor staging and prognosis
Irradiation/chemotherapy	Possible mass size reduction before operation
**Preoperative assessment**	
MDT	Anesthesia, surgery, oncology, radiology, critical care, pathology, *etc*
Risk classification	As shown in Table 2
**Anesthesia management**	
Premedication	Preoperative sedatives should be administered cautiously
Transfer	Transfer of high-risk patients should be accompanied by anesthesiologists
Equipment	Multi-functional operating table is available for prompt intraoperative position change
	Extracorporeal circulation machine should be ready for rescue use
Monitoring and venous access	Arterial line is placed prior to induction
	Femoral vein is preferred, especially in medium to high risk patients
Anesthesia induction	Avoid deep sedation and proceed with stepwise induction Volatile anesthetics, ketamine, dexmedetomidine, remifentanil and propofol are preferred for induction Spontaneous ventilation is preferred Muscle relaxants avoidance is recommended (choose succinylcholine if needed)
Endotracheal intubation	Awake fiberoptic intubation with a reinforced single-lumen endotracheal tube is preferred
**MMS management**	
	Table-tilted to rescue position Sternum-lifting or mass pull-up by surgeon Inotropic support, volume replacement Extracorporeal circulation

CT, computed tomography; FDG-PET, fluorodeoxyglucose positron emission tomography; MDT, multiple disciplinary team; MMS, mediastinal mass syndrome; MRI, magnetic resonance imaging; PFT, pulmonary function test.

## Conclusions

To sum up, pre-operative multi-disciplinary discussion, well-planned anesthetic management and pre-determined protocols for emergency situations are all vital to patient safety.

## Data Availability

The original contributions presented in the study are included in the article/Supplementary Material, further inquiries can be directed to the corresponding author/s.

## Appendix. Search strategy

**Table:**

**PUBMED**
#1	((((((((“mediastin”[All Fields] OR “mediastinally”[All Fields]) OR “mediastine”[All Fields]) OR “mediastinic”[All Fields]) OR “mediastinitis”[MeSH Terms]) OR “mediastinitis”[All Fields]) OR “mediastinum”[MeSH Terms]) OR “mediastinum”[All Fields]) OR “mediastinal”[All Fields]) AND “mass”[All Fields]
#2	“anaesthetically”[All Fields] OR “anaesthetics”[All Fields] OR “anesthetics”[Pharmacological Action] OR “anesthetics”[MeSH Terms] OR “anesthetics”[All Fields] OR “anesthesiology”[MeSH Terms] OR “anesthesiology”[All Fields] OR “anaesthetise”[All Fields] OR “anaesthetised”[All Fields] OR “anaesthetising”[All Fields] OR “anaesthetization”[All Fields] OR “anaesthetize”[All Fields] OR “anaesthetized”[All Fields] OR “anaesthetizing”[All Fields] OR “anesthetic s”[All Fields] OR “anesthetically”[All Fields] OR “anaesthetic”[All Fields] OR “anesthetic”[All Fields] OR “anesthetization”[All Fields] OR “anesthetize”[All Fields] OR “anesthetized”[All Fields] OR “anesthetizes”[All Fields] OR “anesthetizing”[All Fields] OR “anaesthesia”[All Fields] OR “anesthesia”[MeSH Terms] OR “anesthesia”[All Fields] OR “anaesthesias”[All Fields] OR “anesthesias”[All Fields]
#3	(((((((((((“manage”[All Fields] OR “managed”[All Fields]) OR “management s”[All Fields]) OR “managements”[All Fields]) OR “managing”[All Fields]) OR “managment”[All Fields]) OR “organization and administration”[MeSH Terms]) OR (“organization”[All Fields] AND “administration”[All Fields])) OR “organization and administration”[All Fields]) OR “management”[All Fields]) OR “disease management”[MeSH Terms]) OR (“disease”[All Fields] AND “management”[All Fields])) OR “disease management”[All Fields]
#4	“case reports”[Publication Type] OR “case report”[All Fields]
#5	#1 AND #2 AND #3
#6	#1 AND #2 AND #4
#7	#5 OR #6
**EMBASE**	
(“mediastinal mass”/exp OR “mediastinal mass” OR [mediastinal AND (“mass”/exp OR mass)] AND ((“anesthesia”/exp OR anesthesia) OR (“anesthetic”/exp OR anesthetic)) AND “management”/exp OR management) AND (“mediastinal mass”/exp OR “mediastinal mass” OR [mediastinal AND (“mass”/exp OR mass)] AND ((“anesthesia”/exp OR anesthesia) OR (“anesthetic”/exp OR anesthetic)) AND “case repor”'/exp OR “case report” OR (case AND report))	
**OVID**	
#1	mediastinal mass.ti,ab,tx.
#2	anesthetic.ti,ab,tx. OR anesthesia.ti,ab,tx.
#3	case report.ti,ab,tx.
#4	#1 AND #2 AND #3
**CNKI**	
(SU = “纵隔肿瘤” OR SU = “纵隔占位”) AND (SU = “麻醉管理” OR SU = “麻醉经验” OR SU = “麻醉方法”) OR (SU = “纵隔肿瘤” OR SU = “纵隔占位”) AND (TI = “病例报道” OR AB = “病例报道” OR KY = “病例报道”)	
